# Impacts of COVID-19 on Patients With Common Surgical Emergencies at the King Fahad Specialist Hospital in Buraidah, Saudi Arabia

**DOI:** 10.7759/cureus.31868

**Published:** 2022-11-24

**Authors:** Sultan Alsaigh, Mohammad Harisi, Reem Almuhaymidi, Abdullah A Al-Hojailan, Abdulaziz Z Alharbi, Shahad S Alolayan, Razan S Alqarzaee, Ibrahim Algosair

**Affiliations:** 1 General Surgery, King Fahad Specialist Hopsital, Buraidah, SAU; 2 General Surgery, King Fahad Specialist Hospital, Buraidah, SAU; 3 Medicine, Qassim University, Qassim, SAU; 4 Medicine, Qassim University, َQassim, SAU; 5 Medicine, Qassim University, ََQassim, SAU

**Keywords:** hernia, surgical emergencies, acute calculus cholecystitis, acute appendicitis, covid 19

## Abstract

Introduction: The COVID-19 pandemic is a global disaster with millions of infections and deaths. Healthcare systems and services were significantly affected, necessitating adjustments. These included postponement of scheduled appointments and elective surgeries. During the pandemic, there was an increase in the number of acute appendicitis, gallstones, and hernia with a significant impact on the signs and symptoms of presenting problems due to prehospital delay.

Aim: This study aims to measure the impacts of COVID-19 on patients with common surgical emergencies in King Fahad Specialist Hospital, Buraidah, Saudi Arabia.

Methods: This is a single-center retrospective study conducted at King Fahad Specialist Hospital in Buraidah, Saudi Arabia. We reviewed all medical records of patients diagnosed with common surgical emergencies (acute appendicitis, gallstones, and hernia) during a selected time of COVID-19 lockdown and compared it with a similar set period before the crisis as a control sample. All medical records were reviewed to find out the overall number of admissions, frequency of emergency department (ED) visits, duration of illness, picture of clinical presentation, intraoperative findings, course and duration of admission, and final pathology if any.

Results: A total of 322 patients were included in the study. Of these, 119 (37%) patients underwent surgery before COVID-19 while 203 (63%) patients underwent surgery during the pandemic. The diagnosis of acute appendicitis was 63.9% and 47.7%, hernia 27.7% and 34.6%, and gallstone was 8.4% and 17.7% for control and pandemic periods, respectively. The duration varied from 10 hours to two days and four hours to one month, seven hours to one day to eight hours to six months, and two hours to one day to seven hours to one and half a month for acute appendicitis, hernia, and gallstone in control and pandemic period, respectively. The mean length of stay for acute appendicitis was reduced from two days during the control period to one day during the pandemic period, from four to three days for gallstone, and for hernia, it remained three days for both the control and pandemic periods, respectively. Regarding the course of admission for acute appendicitis, the uneventful cases were reduced while an increase in uneventful cases for both hernia and gallstone was observed.

Conclusion: During the COVID-19 pandemic, there was a noticeable reduction in hospital visits. We observed an increase in the number of one-time visits and a reduction of three, four, and seven-time visits, which was attributed to the fact that patients have been reported to visit the hospital after a long time from the onset of symptoms with a higher chance of complication and subsequent surgeries. The number of acute appendicitis cases was reduced while the cases of hernia and gallstones increased significantly. The minimum period for the duration of acute illness for appendicitis was reduced in the pandemic period, while the minimum period for both gallstone and hernia was increased as both conditions could require conservative management. The mean length of hospital stay was reduced during the pandemic period, mainly due to the early discharge implemented in COVID-19 protocols to decrease the risk of infection. The severity of symptoms was increased due to the cancellation and delaying of surgeries.

## Introduction

A virus is an obligate intracellular infectious unit that targets cells susceptible and permissive to its life cycle [1]. Coronaviruses (CoVs) belong to the C*oronaviridae *family and are enveloped viruses containing a positive single-stranded RNA. Recently, a novel coronavirus caused a major worldwide threat to public health after it originated in Wuhan, Hubei Province, China, in December 2019 [2]. The WHO officially announced and assigned it the name COVID-19 on 11 February 2020. This disaster has affected most countries worldwide, causing millions of infections and deaths [2]. Furthermore, directly, or indirectly, healthcare systems and services were significantly affected by COVID-19, necessitating adjustments and amends. These included postponement of scheduled appointments and elective surgeries. Additionally, it affected patients' behavior regarding their visits to healthcare providers due to their fear of infection risk and a hesitance to burden the system [3]. 

The rapid spread and high transmissibility reached Saudi Arabia, with the first documented case in a traveler returning from Iran on March 2020. Saudi Arabia was among the first countries to start preparations for the pandemic, and multiple preventive and precautionary measures were taken to control the spread of COVID-19. Additionally, continuous efforts were made by the Saudi government to minimize the impact of COVID-19 and prevent it from further spreading, which included suspending all religious activities and shifting the educational process to remote learning and virtual classrooms to ensure the safety of the population [4].

Fear of infections and implementation of stay-at-home precautions have affected many surgical emergencies. Surprisingly, the number of surgical emergencies significantly increased along with an increase in the rate of complications [5]. A multicentric retrospective cohort study was conducted in Spain, including three hospitals and a sample of 402 patients. They compared the patients who underwent acute surgical care before and during the pandemic. A significant reduction (58%) in the number of patients seeking healthcare providers and acute surgical interventions was found [6]. 

One of the most common abdominal emergencies is acute appendicitis. Open or laparoscopic appendicectomy is the treatment of choice [7]. A retrospective study was conducted at Baystate Medical Center (BMC), western Massachusetts, USA, investigating the management of acute appendicitis during the pandemic. Results showed an increase in the rate of complicated appendicitis in comparison to the previous two years [8]. Additionally, a retrospective study was done in Brazil comparing two groups who underwent appendectomy during March and April 2020 and the same months in 2019. A significant reduction in the number of appendectomies during the 2020 period compared to the 2019 period (57%) was found. Moreover, results show a significant increase in the rate of complicated appendicitis (33.3% vs. 15.2%, p = 0.04) [5]. Another retrospective analysis was carried out in Miami, Florida, USA, in which 48 patients reported acute appendicitis, including 16 patients with perforations, compared to the previous year where 59 patients presented with acute appendicitis with 10 cases of perforated appendicitis (33% vs. 17%, p = 0.04) [9].

The occurrence of gallstones, a common surgical problem, has also been affected by the pandemic [10]. Acute pancreatitis and cholangitis are the well-recognized complications of gallstones, while prolonged irritation may lead to the development of gall bladder carcinoma [11]. There is a noticeable reduction in the presentation of cholecystitis during the lockdown period. A retrospective study conducted in New Zealand reported a reduction of 39.2%. In addition, 52.9% of the patients in the compared group were managed with laparoscopic cholecystectomy as opposed to only one patient over the lockdown period [12]. Similarly, in the UK, the management of gallstone disease during the COVID-19 pandemic (MEGAVID) clinical investigator group reported a decrease in cholecystectomy rates by 72.2% [13].

In addition, an inguinal hernia repair is one of the most performed surgical procedures. Recently, inguinal hernia repair has been modified from pure tissue repair to prosthetic laparoscopic repair [14]. At George Eliot Hospital, UK, a retrospective study showed an 18% increase in emergency hernia surgery operations, with a 23% increase in visceral resections due to irreparable herniated content and strangulation [15].

During the ongoing COVID-19 pandemic, there was a noticeable increase in the number of common surgical emergencies with a significant impact on the signs and symptoms of presenting complaints due to pre-hospital delay. However, such a characterization in Saudi Arabian hospitals has not been performed before. Therefore, this study aims to bridge this gap by measuring the impacts of COVID-19 on patients with common surgical emergencies in King Fahad Specialist Hospital, Buraidah, Saudi Arabia. Specifically, we measured the effect of COVID-19 on patients admitted through the ED with acute appendicitis, gallstone, and hernia from the overall number of admissions, frequency of ED visits, the clinical picture at presentation, duration of acute illness, intra-operative findings, course of admission, and final histopathology if available.

## Materials and methods

Our study design is a retrospective chart review and an analytic study conducted in King Fahad Specialist Hospital, the biggest hospital in Buraidah, the capital of the Al-Qassim region in Saudi Arabia with over 600,000 population (Qassim Region Research Ethics Committee (QREC) issued approval no. 1443-1143546). We reviewed all medical records of patients admitted to the hospital through the ED and diagnosed with common surgical emergencies (e.g., acute appendicitis, gallstones, and hernia) during a selected period of COVID-19 lockdown and compared it with the same selected period before the crises as the control sample. All medical records were reviewed to find out the overall number of admissions, frequency of ED visits, duration of illness, picture of clinical presentation, intraoperative findings, course, duration of admission, and final pathology if any. We included all patients admitted through ED with selected dates regardless of age, sex, and comorbidities. Any patients admitted electively or any changes in diagnosis after admission were excluded from the study. We included all patients who met the criteria in two chosen periods: the covid era from the 1st of September 2020 to the 30th of February 2021 and the pre-covid era from the 1st of September 2019 to the 30th of February 2020, and the total number of patients was 322. After obtaining the information required for the sample group, the data of each diagnosis was compared head-to-head with the control group. The data was collected anonymously without any personal identifiers. The data were retrospectively collected from the medical records followed by the application of inclusion and exclusion criteria. After that, each group of patients with the same diagnosis was divided into two groups (sample and controlled) then we labeled them numerically from one to 322 to keep the identity of the patient anonymous. Lastly, the collected data were entered into Microsoft Excel (Microsoft Corp., Redmond, WA, USA) and transferred to SPSS (IBM Corp., Armonk, NY, USA) for statistical analysis.

## Results

As Table 1 indicates, 322 patients were included in the study. Among those, 119 (37%) patients underwent surgery before COVID -19, while 203 (63%) patients underwent surgery during the pandemic. The minimum age of patients was 12 before and during the COVID-19 periods, while the maximum age was 91 during the control period, and 74 during the pandemic period. Out of the total patients, there were 54 female patients (45.3%), and 65 male patients (54.7%) before the COVID-19 pandemic, while the numbers increased during the pandemic period i.e., 110 females (54.1%) and 93 males (45.9%). Diabetes mellitus, hypertension, hypothyroidism, obesity, and no comorbidities were present in three, four, five, two, and 97 patients, respectively, before the COVID-19 pandemic. Conversely, during the pandemic period, diabetes mellitus, epilepsy, hypertension, hypothyroidism, and obesity were present in five, seven, two, nine, three, and two patients, respectively. And the patients with no comorbidities were 173.

**Table 1:** Comparison of the demographic and clinical history of patients undergoing acute care surgery in the control and pandemic periods

Variables	Before covid		During covid	
Total respondents: 322				
Number of patients	119 (37%)		203 (63%)	
Age				
Minimum	12		12	
Maximum	91		74	
Mean	30.2		34.8	
Gender	Males	65 (54.7%)	Males	93 (45.9%)
	Female	54 (45.3%)	Female	110 (54.1%)
Nationality	Saudi	105	Saudi	180
	Bangladesh	3	Bangladeshi	1
	Indian	3	Indian	3
	Sudanese	3	Sudanese	3
	Pakistani	2	Pakistani	3
	Others	3	Egyptian/others	6/7
Comorbidity	Bronchial asthma (BA)	5	Bronchial athma (BA)	5
	Diabetes mellitus (DM)	3	Diabetes mellitus (DM)	7
	Hypothyroidism	5	Epilepsy	2
	Obesity	2	Hypothyroidism	3
	Pregnant	3	IBS	2
	Hypertension (HTN)	4	Obesity	2
			Hypertension (HTN)	9
	No comorbidities	97	No comorbidities	173

**Figure 1:**
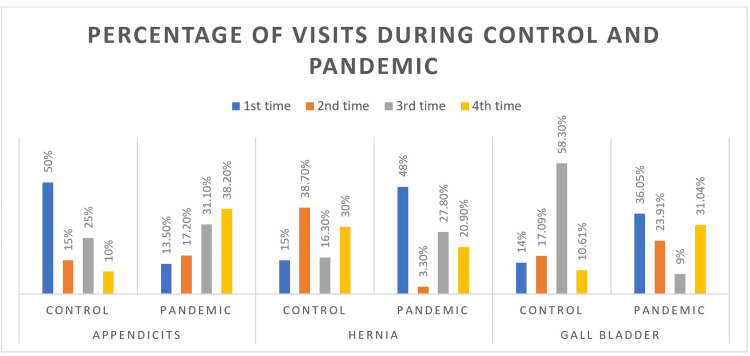
Bar graph comparing the diagnosis before covid and during covid

Figure 1 compares the diagnosis of acute appendicitis, hernia, and gallstone. Before COVID-19, 50% of acute appendicitis patients visited the hospital once to be admitted, and 15 % of patients visited twice. Additionally, 25% of patients visited the hospital for a third follow-up, and 10% of patients visited the hospital for a fourth follow-up. During the COVID-19 period, only 13.50% of patients went to the hospital once. This percentage of visits increased for the fourth visit. Around 17.20% were acute appendicitis patients who visited the hospital for the second time. Those patients who went for a third visit were 31.10%, and this percentage was greater than those who went for the third time before COVID-19. Around 38.20% of patients visited the hospital for the fourth time.

Before COVID-19, there were only 15% of patients with hernias went to the hospital once, and 38.70% of patients visited a second time. The patients who visited a third time were 16.30%, and 30% visited a fourth time. During the pandemic, the maximum number (48%) of patients with hernia visited the hospital once as compared to the control group. A minimum of 3.3% of patients visited a second time. Around 27.80% visited a third time, and 20.90% of these patients visited a fourth time. 

In the case of gallstones, 14% of patients went to the hospital for the first time before COVID-19, and 17.09% visited a second time. The maximum number of people with gallstone visited the hospital for the third time (58.30%). Around 10.61% of patients visited the hospital for the fourth time before COVID-19. If we compared these percentages of patients to those percentages of patients during COVID-19, 36.05% of patients visited the hospital once while 23.91% went a second time. Only 9% of patients visited the hospital for the third time, and 31.04% visited the fourth time.

Table 2 explains the diagnosis of acute appendicitis (3.9% and 47.7.1%), hernia (27.2% and 34.6%), and gallstone (8.4% and 17.7%) for the control and pandemic periods, respectively. The duration of the disease varied from 10 hours to two days in the control period and four hours to one month in the pandemic period for acute appendicitis; seven hours to one day in the control period, and eight hours to six months in the pandemic period for hernia; and two hours to one day in the control period to seven hours to 1.5 months in the pandemic period for gallstone. The mean length of stay shows that the average length of stay for acute appendicitis was reduced from two to one day during the COVID-19 pandemic, from four to three days for gallstones, and three days in both periods for hernias. Regarding the course of admission, for acute appendicitis, the uneventful cases were reduced from 85.4% in the control period to 70.6% in the pandemic period, while an increase in uneventful cases for both hernia and gallstone was also observed. 

**Table 2:** Comparison of surgical history of patients undergoing acute care surgery in the control and pandemic period

Variables		Acute Appendicitis		Hernia		Gallstone	
		Control group	Pandemic group	Control group	Pandemic group	Control group	Pandemic group
Diagnosis (hernia, gallstone, or acute appendicitis)		76 (63.9%)	97 (47.7%)	33 (27.7%)	70 (34.6%)	10 (8.4%)	36 (17.7%)
Duration of acute illness	2 days to 10 hours		1 month to 4 hours	1 day to 7 hours	6 months to 8 hours	1 day to 2 hours	1.5 months to 7 hours
Mean length of stay (days)		Average stay 2 days and 6 hours	Average stay 1 day and 4 hours	Average stay 3 days and 8 hours	Average stay 3 days	Average stay 4 days and 8 hours	Average stay 3 days and 5 hours.
Course of admission		Uneventful: 85.4% Post-op drain: 14.6%	Uneventful: 70.6% Post-op drain: 29.4%	Uneventful: 74.03 Post-op drain: 25.9%	Uneventful: 80.5% Post-op drain: 19.5%	Uneventful: 56.6% Post-op drain: 43.4%	Uneventful: 68.34% Post-op drain: 31.66%

In Table 3, the quantitative variables show the frequencies of acute care surgeries in the control and pandemic period as the mean ages were 30.2 (12 to 91). This included 45.3% females and 54.7% males before COVID-19 while the mean ages were 34.8 (12 to 74) out of which the female ratio was 54.1%. The males were 45.9% of the study population during the pandemic era. The diagnosis of hernia, acute appendicitis, and gallstones were 6%, 63.9%, and 17.2%, respectively, before COVID-19. During COVID-19, the percentages were 94%, 36.1%, and 82.8%, respectively.

**Table 3:** Quantitative variables of patients undergoing acute care surgery in the control and pandemic period as percentage and p-value

	Control	Pandemic	p-value
Mean age (years)	30.2 (12-91)	34.8 (12-74)	0.255
Gender (% female)	45.3%	54.1%	0.002
Gender (% Male)	54.7%	45/9%	0.001
Mean length of stay (hours)	75	55	0.001
Hernia %	6%	94%	0.001
Acute appendicitis %	63.9%	36.1%	<0.001
Gallstone %	17.2%	82.8%	<0.001

Diagnoses changed during the pandemic; acute appendicitis, hernia, and gallstones had different results. The visits for acute appendicitis decreased during the pandemic period, and an increase in visits was observed for hernia and gallstones.

## Discussion

With the onset of the COVID-19 pandemic, several studies have been conducted to explore patients' surgical experiences during and before COVID-19 [16,17]. Several factors were investigated, such as patients' stay during a pandemic, psychological issues due to lockdown, and infectious causes in the pandemic era. Still, as far as we know, the number of patients for acute care medical surgical emergencies has not been previously studied in Saudi Arabia [18-20]. Our study was conducted in the King Fahad Hospital of Saudi Arabia to measure the effects of COVID-19 on the overall number of admissions, frequency of ED visits, duration of acute illness, and intra-operative findings, and course of admission.

The number of acute care surgeries for acute appendicitis, hernia, and gallstone increased from 37% to 63% during the pandemic, doubling the activity that occurred before COVID-19. The increase in the number of surgical cases should be carefully considered in those areas where COVID-19 is still developing or there is a risk of another infectious outbreak to improve the management of surgical patients. The resources are usually oriented to the infected patients with COVID-19, ultimately increasing the number of surgical issues after surgery in the emergency department. While the frequency of ED visits was disrupted by 3% because 72.2% of patients visited once before COVID-19, only 0.4% of patients had ED visits to the King Fahad Hospital of Saudi Arabia four or seven times. The increase in the number of one-time visits and reduction of three, four, and seven-time visits was caused due to several factors. Among them, the commonest factor was that the patients visited the hospital after the onset of severe symptoms such as vomiting, constipation, swelling, migraine, nausea, anorexia, etc.

The significant delay between the patient's arrival and clinical presentation of symptoms has also been reported in another emergency department due to the fear of developing hospital-acquired infection and the lack of medical supplies [21,22]. It has been estimated this delay could lead to more severe outcomes, such as the development of gallbladder spillage or rupture [23-25].

We observed that 63.9% of acute appendicitis were reported in the control period while 47.7% were reported in the pandemic period. The change in ED visits for acute appendicitis is expected to be insignificant or decreased due to several reasons. First, unlike gallstones and hernias, which could be managed conservatively, acute appendicitis is considered a surgical emergency and needs urgent intervention. Second, the resources are usually oriented to the infected patients with COVID-19, as explained earlier. Third, the rate of medical management of acute appendicitis has increased significantly during the pandemic period. Fourth, the surgeon’s threshold to admit acute appendicitis patients was lower during the pandemic than before. The percentage of emergency surgeries for hernia and gallstones increased from 27.7% to 34.6% and 8.4% to 17.7% in the control and pandemic periods, respectively. The reason behind this significant increase is the holding of elective admissions through outpatient departments for these cases. The increase in visiting patients during the pandemic can also be explained due to the modifications in the lifestyle of patients during the COVID-19 lockdown [26]. For instance, during the COVID-19 lockdown, physical activities had been decreased while the dietary fat quality and quantity increased, resulting in complications of gallstones, acute appendicitis, and hernia. Other conservative therapies could also treat these diseases [27-29]. But these therapeutic options could increase the patient's visits, resulting in the chances of COVID-19 infection and post-COVID-19 complications [30].

When the presence of comorbidities was considered for the control and pandemic periods, it was concluded that the number of patients with comorbidities was higher in the control period (18.48%) than in the pandemic period (14.77%), entailing that it has no significance in the contribution to surgical presentations within our study group.

When the duration of acute illness was analyzed, it was concluded that the minimum period for acute appendicitis was reduced from 10 hours during the control period to four hours during the pandemic time due to late presentation. Conversely, the duration of acute illness for both gallstone and hernia increased significantly as both conditions could be managed conservatively.

The mean length of hospital stay was also reduced for different reasons, mainly early discharge implemented in COVID-19 to decrease the risk of no-social infection. Finally, it was concluded that the development of complications was more common during the pandemic, resulting in reduced patient visits, staying periods, and duration of acute illness.

There were many limitations in the study. First, the provision of data was unsatisfactory. Second, post-surgery complications such as bleeding and post-operative ileus were not investigated. Third, this research does not examine the patients who underwent non-operative treatment. In addition, the patients during the pandemic period who developed COVID-19 infection were not recruited in the datasheet. The major strength of this paper is that it was performed in the central hospital system of Saudi Arabia with accurate data sheets and comprehensive patient information.

## Conclusions

During the COVID-19 pandemic, there was a significant reduction in visits to the hospital. We observed an increase in the number of one-time visits within the pandemic period, and a reduction of three, four, and seven-time visits. This observation may be due to several factors. For example, patients reported visiting the hospital after a long time from the onset of symptoms which exposed them to a higher risk of developing complications and meeting the indications for operations from their first visit. The number of patients with comorbidities was higher in the control than in the pandemic period, which implies that it had no significance in the contribution to surgical presentations within our study group. The number of acute appendicitis cases was reduced during the pandemic period. In contrast, the cases of hernia and gallstones increased significantly. When the duration of acute illness was analyzed, the minimum period for acute appendicitis was reduced in the pandemic period, while the minimum period of acute illness for both gallstone and hernia increased, as both conditions could be managed conservatively initially. The mean length of hospital stay was reduced during the pandemic period for different reasons, mainly due to early discharge as per COVID-19 protocols to decrease the risk of infection. Lastly, the severity of symptoms increased due to the cancellation of schedules and delaying of surgeries.
